# Proteomic profiling identifies candidate biomarkers for small intestinal neuroendocrine tumours G1 and G2

**DOI:** 10.1111/jne.70289

**Published:** 2026-10-09

**Authors:** Malin Grönberg, Dimitrios Papantoniou, Eva Tiensuu Janson

**Affiliations:** ^1^ Department of Medical Sciences, Endocrine Oncology Uppsala University Uppsala Sweden; ^2^ Department of Oncology Ryhov County Hospital Jönköping Sweden; ^3^ Department of Medical Sciences, Endocrine Oncology and Science for Life Laboratory Uppsala University Uppsala Sweden

**Keywords:** neuroendocrine neoplasia, olink, small intestinal neuroendocrine tumours, tumour biomarkers

## Abstract

Although small intestinal neuroendocrine tumours (SI‐NET) Grade 1 (G1) and 2 (G2) are often studied together, clinical course and response to treatment may differ. We hypothesized that these tumour groups express different markers in blood that may inform future treatment strategies. Blood from 107 SI‐NET patients (G1 n = 64, G2 n = 43) and 50 healthy controls was analysed with proximity extension assay (Olink, Uppsala, Sweden). Two oncology protein panels were used. Outcomes were analysed with regression analysis and machine learning algorithms (Random Forest, Boruta). In age‐adjusted analyses, 188 proteins differed between patients and healthy controls, with most being elevated in patients. Six proteins were elevated in G2 vs. G1 patients. After adjusting for age, Retbindin (RTBDN), Cryptic family protein 1 (CFC1), and Calbindin 1 (CALB1) remained significant, while adjusting for age and stage left only RTBDN significant. In a stage IV‐restricted analysis, RTBDN was higher in G2 than in G1 (estimate 1.688; 95% CI 0.796–2.580; *p* <0.001). RTBDN and Midkine (MDK) were associated with poor survival, whereas the opposite was seen for Integrin subunit alpha V (ITGAV). RTBDN emerged as the only candidate biomarker associated with tumour grade after adjustment for age and stage in the G1/G2 analysis. In the stage IV‐restricted analysis, RTBDN remained the top‐ranked protein and showed a similar direction of association without reaching the strict Bonferroni‐adjusted significance threshold. Higher RTBDN levels were also associated with increased mortality risk. These findings are exploratory and require independent validation before clinical application.

## Graphical abstract

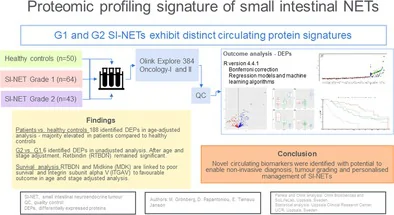

## 1 INTRODUCTION

Neuroendocrine neoplasms (NENs) are a group of tumours that arise from neuroendocrine cells found in various organs. According to the WHO classification, well‐differentiated neuroendocrine tumours (NETs) are categorized into grades based on their proliferation rate and sub‐grouped as G1 (Ki‐67 <3%), G2 (Ki‐67 3–20%), and G3 (Ki‐67 >20%) NET. A fourth group consists of rare, poorly differentiated neuroendocrine carcinomas (NEC, Ki‐67 >20%).^1^ Tumours are further classified using the tumour‐node‐metastasis (TNM) staging system to stage I‐IV, which assesses the extent of tumour invasion and spread.^2^ Small intestinal neuroendocrine tumours (SI‐NETs) are rare, typically slow‐growing tumours, and can be associated with prolonged survival even when metastasized.^3^ SI‐NETs originate from enterochromaffin cells which synthesize and secrete a range of bioactive amines and peptides, including, for example, serotonin and tachykinins. Increased levels of these factors may lead to the carcinoid syndrome with distinct clinical features.^4^ Accurate diagnosis of SI‐NETs is often delayed by several years, by which time many patients present with metastatic disease.^3^

Tumour‐secreted amines and proteins may serve as biomarkers for both diagnosis and longitudinal monitoring. The biomarkers most commonly used today in the diagnosis and follow‐up of SI‐NETs are chromogranin A (CgA) and 5‐hydroxyindoleacetic acid (5‐HIAA), the breakdown product of serotonin. While useful for detecting NETs of any origin, CgA lacks specificity, since it may also be elevated in various non‐tumour‐related conditions (e.g., heart and renal failure, cardiac and inflammatory diseases, prostate cancer and the use of proton pump inhibitors).^3^ The NETest is a multianalyte PCR‐based assay that measures neuroendocrine tumour gene expression in blood. However, its place and usefulness in clinical work‐up is still to be determined.^5^, ^6^

Proximity extension assay (PEA) technology has been employed in numerous cancer studies to identify novel biomarker candidates across various tumour types including NET. One study from *2017* discovered new proteins in stage III‐IV SI‐NETs that correlated with survival.^7^ Another study using PEA and the Olink Proseek Oncology II panel, which includes 92 cancer‐related plasma proteins, in combination with machine learning, proposed a multi‐biomarker strategy that may aid in the early detection of SI‐NET.^8^ Using the same Olink Proseek Oncology II panel, a further study reports a multi‐plasma protein strategy that enhances the diagnostic accuracy of pancreatic NETs and distinguishes them from SI‐NET.^9^ However, so far, no new biomarkers have been implemented into clinical use.

Within the SI‐NET group G1 tumours are four times more common than G2 tumours.^10^ Although both G1 and G2 are classified as well‐differentiated, G2 tumours exhibit more aggressive clinical behaviour compared to G1 and likely have an altered molecular profile.^11^ Most SI‐NET studies include both G1 and G2 tumours, often alongside NETs from other origins, or focus solely on the G1 group. The findings from these studies are likely more applicable to the larger G1 group, and it remains uncertain whether results can be extrapolated to the G2 subgroup.

Our hypothesis is that differences in protein expression between G1 and G2 SI‐NET will lead to the discovery of new molecular biomarkers. This will pave the way for identifying key dissimilarities important to understand tumour progression and aggressiveness, which can be valuable in developing new diagnostic tools and treatment strategies. Understanding biological differences can aid in selecting treatment and predicting patient outcomes.

## 2 MATERIALS AND METHODS

### 2.1 Clinical data and samples

Serum samples were prospectively collected from NET patients within 1 month after the date of diagnosis prior to initiation of any disease‐specific treatment, and stored at −80°C. In the present study, we included 110 patients with SI‐NET for analysis. Haemolytic samples (*n* = 3) with pink or red discoloration were excluded by visual inspection. and the remaining 107 patients were included, 64 with G1 and 43 with G2 tumours. Serum from 50 healthy blood donors was used as controls and was provided from 3H Biomedical AB (Uppsala, Sweden). These samples were accompanied by information of gender and age. Each unit was collected in a QA controlled and Certificated Blood Center and was tested and confirmed negative for HBsAg, anti‐HIV1/2, anti‐HCV, and syphilis. Ethical approval for the study was granted, and the need for new informed consent was waived for all participants by the ethical committee in Uppsala 2017/403. All procedures were performed in accordance with the 1964 Helsinki Declaration and its later amendments. Patients provided written consent at the time of treatment for the use of samples and clinical data in retrospective research projects.

### 2.2 Olink proteomics based on proximity extension assay (PEA) technology

Samples underwent a single freeze–thaw cycle prior to initial analysis, during which appropriate volume needed for analysis was pipetted before samples were immediately refrozen. To reduce systematic bias, all samples were randomly distributed both across and within the 96‐well plates. Each plate contained 88 study samples, while the remaining wells were allocated to external control samples. Sample plates were frozen and transported on dry ice to SciLifeLab (RRID:SCR_014078, Uppsala, Sweden) and stored at the facility until analyzed. The samples were analyzed utilizing the Olink panels Explore 384 Oncology‐I and Oncology‐II (Olink Biosciences, Uppsala, Sweden). All included proteins of the panels (368×2) as well as the analytical method can be found at https://www.olink.com/. The analysis was performed by SciLifeLab Uppsala, Sweden.

### 2.3 Statistical analysis

Statistical analysis was performed by Uppsala Clinical Research Center, UCR, Uppsala, Sweden. Two or more groups were compared with unadjusted and adjusted linear regression models. As a sensitivity analysis, the comparison between G1 and G2 was repeated in patients with stage IV disease only. Overall survival (OS) was defined as the time from diagnosis to death from any cause. Patients without a recorded event were censored at the date of last known follow‐up or on 2024‐09‐23, whichever occurred first. Survival was graphically presented using the Kaplan–Meier method, and associations with clinical and molecular variables were assessed using unadjusted and adjusted Cox‐regression models. Regression model results were further compared to variables selected using the Boruta algorithm, a machine‐learning feature selection method built around Random Forest (RF). Boruta identifies all relevant variables in a dataset, by comparing their importance to random “shadow” features, which are permuted copies of the original variables. It aims thus to reduce the risk of spurious predictors, correct for randomness, and account for potential interactions between variables.^12^ Briefly, Boruta iteratively runs RF analyses comparing all variables with the “shadow” features, and classifying variables as confirmed, rejected or tentative depending on their importance compared to the shadow features. Volcano plots were used to visualize the differential expression of proteins in the G1 vs. G2 and tumour vs. healthy controls groups. A stringent Bonferroni correction was used, adjusting for 720 tests per analysis.

Statistical analyses were performed using R version 4.4.1 (RRID:SCR_001905) using standard packages except for the package ranger for Random Forest analyses and Boruta for Boruta analyses.^12^, ^13^, ^14^

### 2.4 Data availability

Raw data for this study were generated at SciLifeLab, Uppsala, Sweden. Derived data supporting the findings of this study are available from the corresponding author upon reasonable request.

## 3 RESULTS

Among the healthy controls, 23 (46%) were male and the median age was 51 years (IQR 45–55.8). Among the patients, 64 (60%) were male and the median age at diagnosis was 64.3 years (IQR 58.4–71.8). Patient characteristics are summarized in Table 1.

**TABLE 1:** Clinicopathological characteristics of SI‐NET patients included in the assay.

	Grade 1	Grade 2
Total, *n*	64	43
Age, years	63.5 (57.1–69.2)	68.8 (61.3–74.5)
Sex, male (%)	39 (61)	25 (58)
Stage		
I	3 (5)	0 (0)
II	3 (5)	2 (5)
III	28 (44)	4 (9)
IV	30 (47)	37 (86)

*Note*: Data are presented as *n*, *n* (%) or median (interquartile range).

Abbreviation: SI‐NET, small intestinal neuroendocrine tumour.

The following 16 assays did not meet Olink's batch release quality control criteria and are therefore not included in this project: Explore 384 Oncology I assay: FLI1, MPI, EBI3 IL27, and ANGPTL7; and Explore 384 Oncology II assay: CPLX2, TAGLN3, GABARAPL1, NFKB2, CTAG1A, CTAG1B, OGT, MTHFSD, IFIT1, TNPO1, MAGEA3, SH3GL3, and RAPGEF2. Criteria included: (1) at least 500 counts per specific combination of sample and assay, (2) the deviation of the median of the negative controls must be ≤5 standard deviations from the set predefined value.

### 3.1 Protein levels in patients compared to healthy controls

244 proteins passed the Bonferroni threshold in an unadjusted analysis, including 150 up‐regulated and 94 down‐regulated proteins in patients compared to healthy controls. After adjusting for age, 188 proteins were differentially expressed, with 117 upregulated and 71 proteins downregulated in patients compared to controls. Among the topmost differentially expressed proteins identified between the patients and healthy controls, Carboxylesterase 2 (CES2), IQ motif‐containing GTPase‐activating protein (IQGAP2), Metastasis suppressor protein 1 (MTSS1), and Mesothelin (MSLN) showed levels significantly lower in patients, whereas DnaJ Heat Shock Protein Family (Hsp40) Member B1 (DNAJB1), Cryptic family protein 1 (CFC1), and Hydroxymethylbilane synthase (HMBS) showed significantly higher levels in patients. Figure 1A,B show the respective volcano plots, highlighting the significant results after correction for multiple testing. Table 2 shows the data for the top proteins (highest significance) in the unadjusted and adjusted analyses.

**FIGURE 1:**
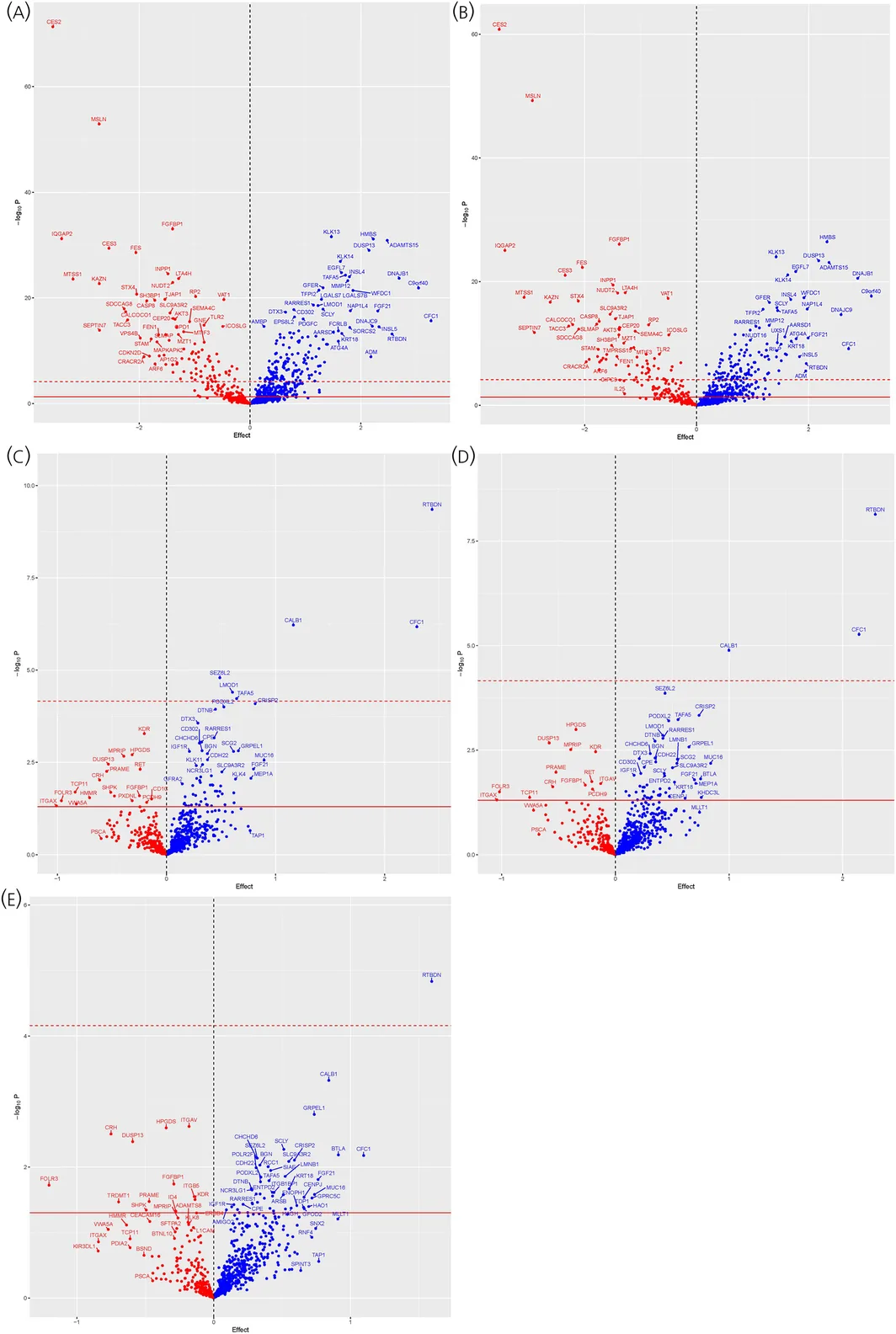
Volcano plot for the analyses of cases vs. healthy controls (A, unadjusted; B, adjusted by age) and G2 vs. G1 respectively (C, unadjusted; D, adjusted by age; E, adjusted by age and stage). Red markers displayed left from the y‐axis are reduced; blue markers displayed on the right side are increased in samples of patients and G2 patients respectively. The red line indicates *p* = −log10(0.05) and the red dashed horizontal line indicates the *p*‐value threshold after adjustment for the total number of tests according to Bonferroni.

**TABLE 2:** Top oncology proteins passing Bonferroni with lowest *p*‐values in patients vs. healthy controls (unadjusted and adjusted analyses) and G1 vs. G2 (unadjusted and adjusted analyses).

Patients vs. healthy controls	Estimate	CI	*p*‐value	Observations below LOD (%)	Boruta status	Median importance
Assay/protein						
Unadjusted						
CES2	−3.558	[−3.769, −3.347]	<1e‐50	0	Confirmed	35.75
MSLN	−2.721	[−2.945, −2.497]	<1e‐50	0	Confirmed	34.08
FGFBP1	−1.396	[−1.571, −1.221]	8.93e−34	0	Confirmed	29.54
KLK13	1.47	[1.279, 1.661]	2.63e‐32	0	Confirmed	24.03
IQGAP2	−3.399	[−3.844, −2.954]	6.28e‐32	0	Confirmed	23.17
HMBS	2.226	[1.934, 2.518]	7.06e‐32	0	Confirmed	26.68
ADAMTS15	2.479	[2.152, 2.806]	1.27e‐31	0	Confirmed	23.86
CES3	−2.545	[−2.894, −2.196]	4.09e‐30	0	Confirmed	22.32
DUSP13	2.15	[1.852, 2.448]	9.84e‐30	0.6	Confirmed	22.38
FES	−2.058	[−2.346, −1.770]	2.66e‐29	69.4	Confirmed	18.13
Adjusted (age)						
CES2	−3.525	[−3.775, −3.275]	<1e‐50	0	Confirmed	35.75
MSLN	−2.934	[−3.192, −2.676]	5.57e‐50	0	Confirmed	34.08
HMBS	2.327	[1.982, 2.672]	3.75e‐27	0	Confirmed	26.68
FGFBP1	−1.382	[−1.589, −1.175]	9.21e‐27	0	Confirmed	29.54
IQGAP2	−3.423	[−3.951, −2.895]	9.23e−26	0	Confirmed	23.17
KLK13	1.419	[1.193, 1.645]	1.02e‐24	0	Confirmed	23.05
DUSP13	2.18	[1.827, 2.533]	4.13e‐24	0.6	Confirmed	22.38
ADAMTS15	2.364	[1.977, 2.751]	8.24e‐24	0	Confirmed	23.86
FES	−2.041	[−2.383, −1.699]	5.22e‐23	69.4	Confirmed	18.13
EGFL7	1.769	[1.466, 2.072]	2.43e‐22	0	Confirmed	18.91

G1 vs. G2	Estimate	CI	*p*‐value	Observations below LOD (%)	Boruta status	Median importance
Assay/protein						
Unadjusted						
RTBDN	2.436	[1.742, 3.130]	4.46e‐10	0.0	Confirmed	50.56
CALB1	1.164	[0.735, 1.593]	6.02e‐07	0.0	Confirmed	39.33
CFC1	2.297	[1.446, 3.148]	6.71e‐07	0.0	Confirmed	25.61
SEZ6L2	0.489	[0.277, 0.701]	1.61e‐05	0.0	Confirmed	13.67
LMOD1	0.605	[0.329, 0.881]	3.98e‐05	0.0	Confirmed	17.90
TAFA5	0.643	[0.342, 0.944]	5.92e‐05	0.0	Tentative	12.70
Adjusted (age)						
RTBDN	2.287	[1.575, 2.999]	7.32e‐09	0.0	Confirmed	50.56
CFC1	2.143	[1.267, 3.019]	5.42e‐06	0.0	Confirmed	25.61
CALB1	0.999	[0.571, 1.427]	1.30e‐05	0.0	Confirmed	39.33
Adjusted (age & stage)						
RTBDN	1.594	[0.908, 2.280]	1.47e‐05	0.0	Confirmed	50.56

*Note*: Estimate is the estimated mean difference from a linear regression model. All but one of the proteins selected by linear regression were also confirmed by Boruta which selects variables based on the median importance score across multiple iterations of the algorithm.

Abbreviations: CI, confidence interval; LOD, lower limit of detection.

### 3.2 Protein levels in G1 vs. G2 tumours

Six proteins showed significantly different blood levels when comparing G1 and G2 patients in an unadjusted analysis after multiple‐test correction: Retbindin (RTBDN), Calbindin (CALB1), Cryptic family protein 1 (CFC1), Seizure 6‐like protein 2 (SEZ6L2), Leiomodin‐1 (LMOD1), and Chemokine‐like protein TAFA‐5 (TAFA5). All were present in higher levels in G2 patients compared to G1 patients. When adjusting for age, three proteins remained significant: RTBDN, CALB1 and CFC1. When adjusting for both age and disease stage, only one marker, RTBDN, reached statistical significance (Table 2 and Figure 1C–E). In the stage IV‐restricted sensitivity analysis, RTBDN showed higher levels in G2 than in G1 (estimate 1.688; 95% CI 0.796–2.580; p‐value 0.000436), with the direction of effect consistent with the main analysis. In this subgroup analysis, no protein remained statistically significant after Bonferroni correction. The 10 top‐ranked proteins in the stage IV‐restricted analysis are presented in Table S1.

The most interesting markers from the comparisons of G1 vs. G2 tumours vs. healthy controls are summarized in Figure 2. Five markers (CES2, IQGAP2, MTSS1, MSLN, DNAJB1) are similarly expressed in G1 and G2 tumours, but differentially expressed in healthy individuals, whereas for three markers (CFC1, RTBDN, CALB1) the expression gradually increases from healthy controls to G1 to G2 tumours.

**FIGURE 2:**
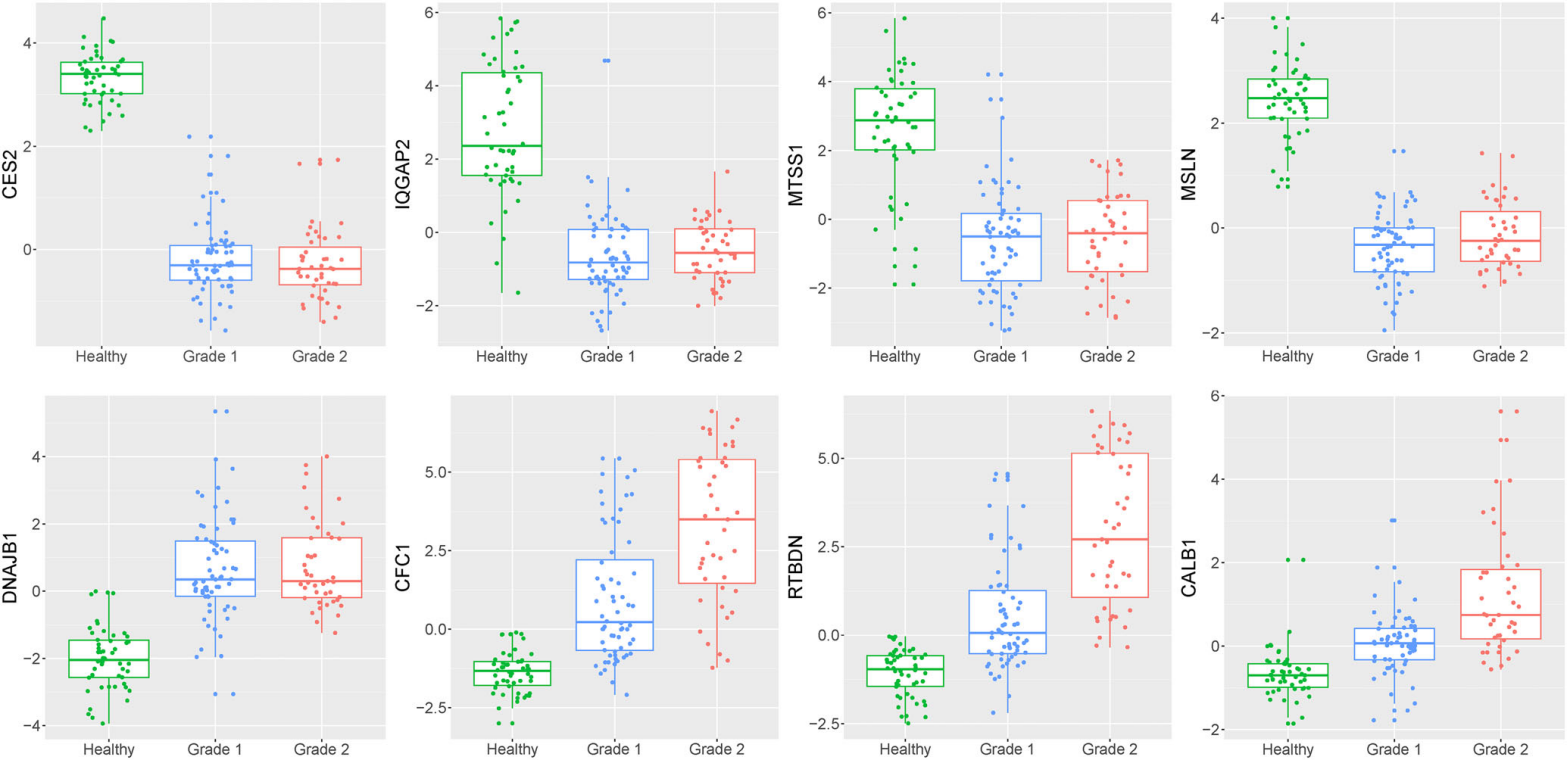
Boxplots of selected markers from PEA analysis.

### 3.3 Variable selection with Random Forest and Boruta algorithms

We further investigated the differential expression of proteins in our cohort using the Boruta algorithm, a feature selection method wrapped around RF. Boruta identified 198 proteins which differ significantly between patients and healthy individuals, and 14 proteins which differ significantly between G1 and G2 tumours. The results are presented in Figure 3 where proteins confirmed as significant are depicted with the green staples. Table 2 indicates which of the top proteins identified with linear regression analysis were confirmed as significant with Boruta.

**FIGURE 3:**
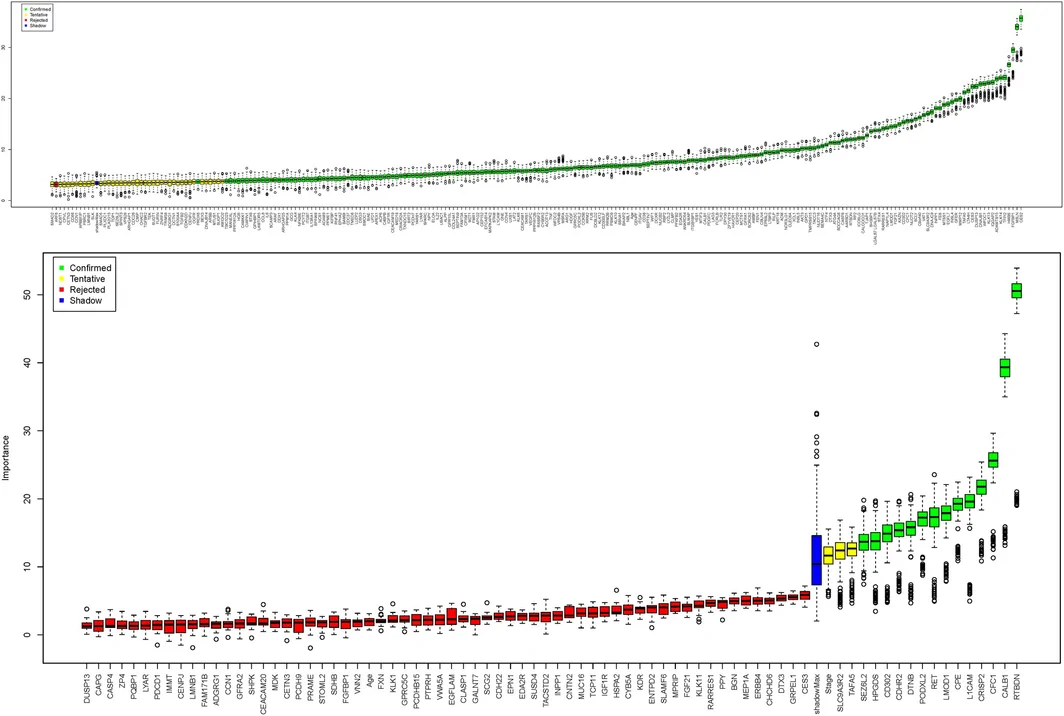
Variable importance in the cohort. Top 240 variables for cases vs. healthy controls (top), and top 80 variables for the outcome G1 or G2 (bottom). Variables are classified as confirmed (consistently more important than random “shadow” features), tentative (borderline importance), or rejected (importance no greater than shadow features).

### 3.4 Survival analysis

During the follow up (median 9.64 years, max 24.08 years) 72 patients died from any cause. Unadjusted Cox‐regression survival analysis revealed that 13 markers passed the Bonferroni threshold including: RTBDN, Tumour necrosis factor receptor superfamily member 27 (EDA2R), CALB1, Kallikrein‐4 (KLK4), LMOD1, Proto‐oncogene tyrosine‐protein kinase receptor Ret (RET), TAFA5, Natural cytotoxicity triggering receptor 3 ligand 1 (NCR3LG1), Podocalyxin‐like protein 2 (PODXL2), SEZ6L2, CFC1, Midkine (MDK) and Matrix metalloproteinase‐12 (MMP12). In an age‐adjusted analysis, four markers passed the threshold: RTBDN, CALB1, Myosin phosphatase Rho‐interacting protein (MPRIP) and CFC1. Finally, in an analysis adjusted for both age and stage, three proteins passed the threshold: RTBDN, MDK and Integrin alpha‐V (ITGAV). Higher RTBDN (HR 1.442; 95% CI 1.246–1.669) and MDK (HR 1.914; 95% CI 1.416–2.587) were associated with an increased, and higher ITGAV with a decreased risk of death (HR 0.161; 95% CI 0.066–0.390). Kaplan–Meier analysis for RTBDN is shown in Figure 4.

**FIGURE 4:**
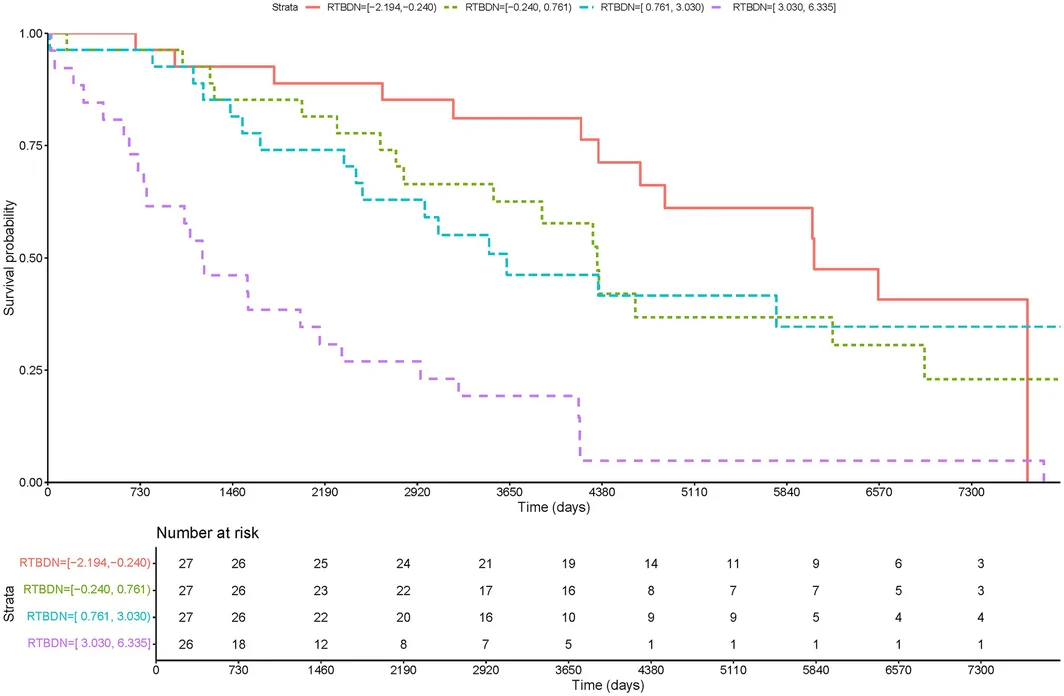
Kaplan–Meier curve for all patients stratified by quartiles for RTBDN.

## 4 DISCUSSION

This study has identified potential novel biomarkers in serum from SI‐NET patients using the Olink PEA method. Unlike most previous studies that included various locations and histopathological grades of NET, this study focused on a comparison of biomarkers between normal healthy individuals, SI‐NET G1 and G2 patients. RTBDN, MDK, and ITGAV proved to be significant discriminators in survival analysis. However, other proteins such as CES2, CFC1, CALB1, and LMOD1 may also be of interest, and their further investigation could be relevant for SI‐NET prognosis/diagnosis and discrimination.

In this study, RTBDN was among the most significant proteins across multiple analyses. RTBDN was first identified in a study of human eye tissues as a retina‐specific protein and shares homology with riboflavin binding proteins. Riboflavin serves as a precursor for the coenzymes flavin mononucleotide (FMN) and flavin adenine dinucleotide (FAD), which play essential roles in intracellular biochemical processes such as energy production, cellular function, growth, and metabolism.^15^, ^16^, ^17^, ^18^ RTBDN has been shown to help concentrate FAD and FMN in the neural retina^19^ and riboflavin, FAD, and FMN levels are reduced in *Rtbdn* ^−/−^ mice.^20^, ^21^ The primary known function of RTBDN is related to retinal health, and currently most studies are linked to this feature.^20^, ^22^, ^23^, ^24^ Although first identified as a retina‐specific protein, data from The Human Protein Atlas (https://www.proteinatlas.org/ENSG00000132026-RTBDN) demonstrate its presence elsewhere as well, and interestingly, in various places linked to the endocrine and gastrointestinal area.

According to RNA expression analysis, RTBDN is enriched in the normal retina as expected, but is also detected in brain, endocrine, and gastrointestinal tissues as well as in several cancer types. Single‐cell RNA analysis shows highest expression in cone receptor cells; however, RTBDN is also detected in enteroendocrine cells. These publicly available data provide biological context for our findings, but do not establish that RTBDN is produced or secreted by SI‐NET cells. Cancer expression analysis further demonstrates that RTBDN is detectable in blood from patients with various cancer types, with the highest levels reported in SI‐NET, myeloma, and paediatric retinoblastoma/sarcoma/neuroblastoma. It is present in higher concentrations in blood from SI‐NET when comparing disease vs. same class disease and vs. other diseases (https://www.proteinatlas.org/humanproteome/blood/Small+intestine+neuroendocrine+tumor). These data provide publicly available evidence that RTBDN is detectable in blood in SI‐NET.

In our study, patients displayed elevated RTBDN concentrations compared to healthy controls. Interestingly, it was also the only protein that remained significant in the adjusted (for age and stage) G1 vs. G2 analysis, with G2 patients having higher levels of the protein compared to G1 patients. Importantly, higher RTBDN levels correlated with worse survival. In the stage IV subgroup analysis, RTBDN remained the top‐ranked protein, with higher levels in G2 compared to G1 patients. The direction and magnitude of the association were broadly consistent with the main analysis, with the observed association below the strict Bonferroni‐adjusted significance threshold. The reason RTBDN is found in SI‐NET patient serum and its possible function is yet unknown. Riboflavin has been associated with increased cancer risk through several mechanisms, including its role in regulating FAD/FMN levels, modulating reactive oxygen species (ROS) production, and influencing apoptotic pathways. Conversely, riboflavin deficiency may exacerbate oxidative stress, further contributing to carcinogenic processes.^16^, ^25^, ^26^ The precise mechanism by which riboflavin is associated with increased risk of cancer has not yet been elucidated. Given that NETs exhibit features of both the nervous and endocrine systems, their shared association with neural tissues may suggest that, upon tumour degeneration, they begin to express a protein typically restricted to retinal neural tissue.

Two other proteins significantly associated with survival in our analysis were ITGAV and MDK. ITGAV is a protein belonging to the integrin receptor family, playing a key role in various cellular processes, particularly in cancer and tissue repair.^27^ Most studies focusing on expression analyses indicate a correlation of ITGAV with tumour progression and poor prognosis.^28^, ^29^, ^30^, ^31^ We found that higher levels of ITGAV correlated with lower mortality risk and with higher levels in healthy controls. It is unclear whether the lower level of ITGAV in patient samples is due to downregulation of ITGAV expression in these tumour cells or linked to other neoplastic regulations leading to altered ITGAV secretion into the blood.

MDK is a multifunctional protein that plays a crucial role in various physiological and pathological processes. MDK overexpression in various tumours results in elevated circulating levels and is associated with poor clinical outcomes across multiple cancer types.^32^, ^33^, ^34^, ^35^ In a PEA study focusing on stage III and IV G1 and G2 SI‐NET patients, MDK exhibited elevated serum concentrations in patients compared to healthy controls.^7^ Similarly, in our study, MDK showed a higher concentration in stage IV G2 patients compared to stage IV G1 patients (p‐value 0.027) but did not pass Bonferroni correction. Higher levels were associated with an increased mortality risk. These results taken together suggest a possible association of MDK and SI‐NET disease severity and poor prognosis.

We found several further proteins differentially expressed between the groups in our study. A panel of these proteins could potentially discriminate G1 from G2 tumours as well as distinguish between those with favourable vs. poor prognosis. This approach might be developed into a liquid biopsy strategy, allowing for the identification of patients with more aggressive SI‐NET without the need of repeated invasive tissue biopsies. Further validation is required to translate these into clinical practice. We are currently exploring more targeted approaches using the data generated in this project.

Our study has several strengths, including the well‐characterized SI‐NET cohort, the inclusion of both G1 and G2 tumours together with healthy controls, prospective collection of serum samples prior to disease‐specific treatment, and the use of stringent multiple‐testing correction combined with complementary machine‐learning‐based feature selection. However, several limitations should be considered when interpreting the findings. First, the substantially higher proportion of stage IV disease in the G2 group represents a potential source of confounding in the G1 vs. G2 comparison. Although we adjusted for disease stage in the multivariable analysis and performed a stage IV‐restricted sensitivity analysis to further assess whether the observed differences could be explained by categorical disease stage, residual confounding related to differences in tumour burden and metastatic extent cannot be excluded. Additionally, restricting the analysis to stage IV disease reduced the cohort to 67 patients, resulting in lower statistical power than the primary analysis; it is thus better interpreted as supportive rather than confirmatory. These findings should therefore be considered hypothesis‐generating and require validation in larger, stage‐balanced cohorts. Second, 16 assays did not meet the predefined Olink quality‐control criteria and were excluded. Although this was necessary to ensure analytical reliability, the exclusion of these assays may have reduced our ability to identify additional biologically relevant proteins. Consequently, potentially relevant biomarkers among the excluded assays could not be evaluated in the present study. Third, the study lacks an independent external validation cohort and orthogonal validation of the Olink measurements using an alternative analytical method, such as ELISA or targeted mass spectrometry. Although Boruta provided a complementary approach to feature selection within the same dataset, it does not replace external validation or establish the reproducibility of the identified associations. Thus, the identified proteins, including RTBDN, should be regarded as candidate biomarkers rather than validated clinical biomarkers. The primary aim of the present study was to identify candidate circulating proteins associated with tumour grade using high‐dimensional proteomic profiling. Accordingly, the study was designed primarily as a discovery study rather than as a biomarker validation study. Finally, although circulating RTBDN showed an association with tumour grade in the primary analysis and with survival, our study does not establish its cellular or tissue origin. We cannot determine whether circulating RTBDN is produced and secreted by SI‐NET cells or reflects a systemic host response to malignancy. Further studies in larger, independent cohorts, including tissue‐based characterization and orthogonal protein measurement, are therefore required to replicate and further investigate these findings, establish the biological source and function of RTBDN, and determine the clinical utility of this candidate biomarker.

In conclusion, our study identified a number of proteins involved in various molecular pathways with differential expression between G1 and G2 SI‐NETs, and between patients and healthy controls. Our findings may offer new insights into the pathogenesis of SI‐NETs. The identified circulating proteins represent candidate biomarkers associated with tumour grade and clinical outcome. Further studies are needed to validate these findings and establish their biological and clinical relevance.

## AUTHOR CONTRIBUTIONS

**Malin Grönberg:** Investigation; writing – review and editing; writing – original draft; visualization. **Dimitrios Papantoniou:** Data curation; writing – review and editing. **Eva Tiensuu Janson:** Conceptualization; data curation; funding acquisition; writing – review and editing.

## CONFLICT OF INTEREST STATEMENT

The authors declare no conflict of interest.

## ETHICS STATEMENT

Ethical approval for the study was granted, and the need for new informed consent was waived for all participants by the ethical committee in Uppsala 2017/403. All procedures were performed in accordance with the 1964 Helsinki Declaration and its later amendments. Patients provided written consent at time of treatment for the use of samples and clinical data in retrospective research projects.

## Supporting information


**TABLE S1.** Top 10 proteins associated with G1 vs. G2 in the Stage IV‐restricted analysis.

## Data Availability

Raw data for this study were generated at SciLifeLab, Uppsala, Sweden. The data that support the findings of this study are available upon reasonable request from the corresponding author. The data are not publicly available due to their containing information that could compromise the privacy of research participants.
