# Supplementary material for: Proteomic profiling identifies candidate biomarkers for small intestinal neuroendocrine tumours G1 and G2
Source: J Neuroendocrinol. 2026 Oct 9;38(10):e70289. doi: 10.1111/jne.70289 (PMC13653069; doi:10.1111/jne.70289)
Supplement: Supplementary file 1 [file JNE-38-e70289-s001.docx]

**Table S1**. Top 10 proteins associated with G1 vs. G2 in the Stage IV-restricted analysis.

| **G1 vs. G2**  **Assay/Protein** | **Estimate** | **CI** | **P-value** | **Observations below LOD (%)** |
| --- | --- | --- | --- | --- |
| **Unadjusted** |  |  |  |  |
| RTBDN | 1.688 | [ 0.796, 2.580] | 0.000436 | 0 |
| CHCHD6 | 0.472 | [ 0.219, 0.725] | 0.000516 | 0 |
| FGFBP1 | -0.351 | [-0.551, -0.151] | 0.001042 | 0 |
| DTNB | 0.486 | [0.208, 0.764] | 0.001045 | 0 |
| GRPEL1 | 0.886 | [0.364, 1.408] | 0.001447 | 0 |
| SLC9A3R2 | 0.713 | [0.284, 1.142] | 0.001786 | 0 |
| CRISP2 | 0.884 | [0.348, 1.420] | 0.001936 | 0 |
| CALB1 | 1.03 | [0.404, 1.656] | 0.001973 | 0 |
| SHPK | -0.847 | [-1.375, -0.319] | 0.002515 | 37.4 |
| TAFA5 | 0.615 | [ 0.219, 1.011] | 0.003328 | 0 |

Estimate is the estimated mean difference from a linear regression model. CI, confidence interval; LOD, lower limit of detection*.*
